# The effect of naltrexone as a carboplatin chemotherapy-associated drug on the immune response, quality of life and survival of dogs with mammary carcinoma

**DOI:** 10.1371/journal.pone.0204830

**Published:** 2018-10-04

**Authors:** Marília Carneiro Machado, João Moreira da Costa-Neto, Ricardo Dias Portela, Mário Jorge Melhor Heine D'Assis, Olindo Assis Martins-Filho, Stella Maria Barrouin-Melo, Natalie Ferreira Borges, Fabiana Lessa Silva, Alessandra Estrela-Lima

**Affiliations:** 1 Department of Anatomy, Pathology and Veterinary Clinics, Federal University of Bahia, Salvador, Bahia, Brazil; 2 Laboratory of Immunology and Molecular Biology, Institute of Health Sciences, Federal University of Bahia, Salvador, Bahia, Brazil; 3 Laboratory of Diagnostic and Monitoring Biomarkers, Research Center René Rachou, Oswaldo Cruz Foundation, Belo Horizonte, Minas Gerais, Brazil; 4 Center for Agrarian, Environmental and Biological Sciences, Federal University of Recôncavo da Bahia, Cruz das Almas, Bahia, Brazil; 5 Department of Agrarian and Environmental Sciences, Estadual University of Santa Cruz, Ilhéus, Bahia, Brazil; Colorado State University, UNITED STATES

## Abstract

The objective of this study was to evaluate the effect of low-dose naltrexone (LDN) as a carboplatin chemotherapy-associated drug in female dogs with mammary carcinoma in benign mixed tumors (MC-BMT) after mastectomy and to assess its association with quality of life and survival rates. Sixty female dogs were included in this study, all of which had histopathological diagnosis of MC-BMT and were divided into three groups: G1 (control), consisting of animals submitted only to mastectomy with or without regional metastasis; G2, composed of treated animals that did not present with metastasis; and G3, treated dogs that presented with metastasis. G2 and G3 were also subdivided according to the treatment administered: chemotherapy alone (MC-BMT(-) C/MC-BMT(+) C) or LDN and chemotherapy (MC-BMT(-) C+LDN/MC-BMT(+) C+LDN). All animals were subjected to clinical evaluation, mastectomy, peripheral blood lymphocyte immunophenotyping, beta-endorphin and met-enkephalin quantification, and evaluation of survival rates and quality of life scores. The results showed higher serum concentrations of beta-endorphin and met-enkephalin, fewer chemotherapy-related side effects, and better quality of life and survival rates in the LDN-treated groups than in LDN-untreated groups (*P* < 0.05). Evaluation of clinical and pathological parameters indicated a significant association between the use of LDN and both prolonged survival and enhanced quality of life. These results indicate that LDN is a viable chemotherapy-associated treatment in female dogs with MC-BMT, maintaining their quality of life and prolonging survival rates.

## Introduction

Canine mammary tumors are very common in small animal clinics, but the exact incidence of malignant neoplasias is difficult to determine since many cases are not brought for veterinary evaluation [1]. However, in a study in Mexico with 1,917 mammary biopsies, 47.5% of cases were confirmed as malignant tumors [2]. Because of this high frequency and the similarity of clinical features between women and female dogs, there is great interest in a comparative study between the two species.

Among malignant mammary tumors in female dogs, mammary carcinoma in benign mixed tumor (MC-BMT) is the most common [3], as seen by a study [4] that found 60% MC-BMT among 51 mammary carcinomas diagnosed in this species. This tumor originates from the malignant transformation of the epithelial component, can present as *in situ* growth or with infiltrating characteristics, as evidenced by a loss of myoepithelial and basal layer continuity, and is associated with invasion of the stroma by cancer cells or complete replacement of the pre-existing benign lesion [5, 6].

Considering the high frequency of this cancer, the high degree of potential malignancy and the lack of specific data in the literature, efforts are being directed to the study of new adjuvant therapies. Primarily, veterinary clinicians are seeking new treatment protocols that can provide higher cure rates, longevity and better quality of life for their patients.

Naltrexone is a synthetic analogue of oxymorphine that is used as an opioid antagonist that binds to the mu, kappa and delta opioid receptors, blocking the effects of endogenous opioids. It is a pure antagonist, meaning that it does has no agonist effects [7]. In human medicine, use of low-dose naltrexone (LDN) as a chemotherapy adjuvant provides increased survival when used to treat some types of cancers [8], including mammary carcinoma. LDN has the capacity to reduce the side effects of chemotherapy and to stimulate the immune system, and it can be used as a single or combined treatment with surgery and chemotherapy [9].

Considering the similarities between mammary tumor features in women and female dogs and the importance of MC-BMT in the latter species, it is important to propose and test new combined treatments for this type of cancer. In this context, the objective of this study was to evaluate the use of LDN as an adjuvant treatment and its effects on the survival and quality of life of dogs with MC-BMT.

## Material and methods

Research protocols were approved by the Ethics Commission for the use of Experimental Animals of the School of Veterinary Medicine of the Federal University of Bahia (protocol n° 10/2013). All procedures listed in this study were conducted according to the guidelines set by the Brazilian College of Animal Use on Experiments (COBEA). All of the animals’ owners provided informed consent and received information on the objective of the study.

### Clinical evaluation and mastectomy

Animals that participated in this study were recruited among dogs that visited the Veterinary Hospital of the Federal University of Bahia (UFBA). Inclusion criteria included a histopathological diagnosis of MC-BMT, the presence of a tumor larger than three centimeters, and no other concomitant disease at the time of evaluation. From 150 animals with mammary neoplasias, 60 female dogs of different breeds were included in this study, with ages ranging from five to 18 years old. Female dogs with MC-BMT were evaluated in a prospective manner and were randomly divided into three groups: G1: (control) composed of animals submitted only to mastectomy, prospectively stratified based on the absence of metastasis (MC-BMT(-)) and with regional metastasis (MC-BMT(+)); G2: treated animals without metastasis; and G3: treated animals with regional metastasis. G2 and G3 were subdivided according to the proposed treatment: carboplatin chemotherapy only (MC-BMT(-) C/MC-BMT(+) C) or carboplatin chemotherapy and LDN (MC-BMT(-) C + LDN/MC-BMT(+) C + LDN). The owners and the veterinarians that evaluated the animals were blinded with respect to which animals were treated with LDN.

All female dogs with mammary neoplasia were submitted to a complete clinical examination, and clinical data were plotted in a specific cancer survey according to the protocol proposed by Ferreira et al. [10]. The preoperative clinical evaluation included peripheral blood sampling for hemogram and serum biochemical profile analysis, thoracic radiological examination (laterolateral right (LLD), laterolateral left (LLE) and ventral-dorsal (VD)), and total abdomen ultrasound to assess for metastasis. Clinical stage classification was performed based on tumor size (T), involvement of regional lymph nodes (N) and the presence or absence of distant metastases (M), based on the TNM system [11]. All animals were subjected to unilateral total mastectomy, with removal of the inguinal lymph nodes, and tumor samples were classified based on histopathological diagnosis according to the World Health Organization (WHO) criteria complemented by the criteria proposed by Cassali et al. [6]. MC-BMT Grade I tumors without metastasis were excluded. Twenty days after surgery, patients from G2 and G3 began chemotherapy. The evaluation period used in this study was two years.

### Naltrexone treatment and chemotherapy

Twenty days after surgery, treatment with naltrexone (LDN) was initiated with an oral 0.1 mg/kg dose every 24 hours for 24 weeks. The animals’ owners were blinded to the purpose of this medication. Every seven days, female dogs were clinically evaluated to assess for potential side effects of the medication, such as lethargy and gastrointestinal disorders (vomiting and diarrhea) [12]. Adverse events were classified as Grade 1 through 5, as previously described by the Veterinary Cooperative Oncology Group´s common terminology criteria for adverse events (VCOG-CTCAE) [13]. The treatment protocol used in this study was based on an experimental protocol previously described for mice, with the same daily frequency, dose and chemotherapy period of administration [9].

During the same period, the chemotherapy sessions began with the use of intravenous carboplatin (300 mg/m^2^) over a five-minute infusion period administered for six sessions at 21-day intervals. Approximately 24 hours prior to each chemotherapy session, hemogram and serum biochemical analyses were performed. Metoclopramide (0.5 mg/kg), ranitidine (2 mg/kg) and promethazine (0.1 mg/kg) were given subcutaneously. Ranitidine (2 mg/kg each 12 hours) and metoclopramide (0.5 mg/kg each eight hours) were orally administered for three days after chemotherapy. All treatment procedures performed outside of the naltrexone intervention were in accordance with the guidelines described by the Brazilian College of Animal Experimentation (COBEA).

### Immunophenotyping of peripheral blood leukocytes

To evaluate the cellular immune response in study animals, 4 mL blood were collected fifty minutes before mastectomy and after the end of chemotherapy treatment (six months after surgery). Blood was collected into 5 mL sterile disposable syringes via jugular venipuncture and then transferred into sterile tubes containing EDTA. Hematological parameters were obtained using an automated blood cell analyzer (ADVIA 60, Bayer HealthCare, Tarrytown, NY, USA).

Immunophenotyping analyses of canine peripheral blood were performed by flow cytometry as described by Araujo et al. [14] and Estrela-Lima et al. [15] to analyze CD3+, CD4+ and CD8+ T-cells counts in the peripheral blood of study animals before and after proposed treatments. Flow cytometry measurements were performed on FACSCalibur equipment (Becton Dickinson, San Jose, CA, USA). The Cell-Quest software package was used for both data acquisition and analysis. Each analysis was performed in duplicate.

### Quantification of serum beta-endorphins and met-enkephalins

Serum samples were obtained from blood collected 50 minutes before and six months after mastectomy and were used for beta-endorphin and met-enkephalin quantification, employing commercial canine-specific ELISA kits (MyBioSource, San Diego, CA, USA). All procedures were performed following the manufacturer´s instructions.

### Assessment of quality of life and survival monitoring

During chemotherapy treatment and follow-up evaluations, assessment of quality of life was performed in all female dogs. Owners completed a questionnaire every 15 days during chemotherapy treatment and follow-up time. The questionnaire was developed as recommended by Yazbek and Fantoni [16] and consisted of 12 questions with four possible response alternatives, where each question ranged from zero to three points, reaching a total of 36 points. A score of zero was considered the worst quality of life and 36 the best. Questions focused on behavioral information, interaction with the owner, and assessment of pain, appetite, sleep disturbances, as well as the presence of vomiting, diarrhea, urinary incontinence or repletion.

All animals included in this study were screened monthly by a staff of veterinarians experienced with oncology when clinical and laboratory tests and thoracic radiological examinations were performed. Owners were instructed to contact the veterinary staff responsible for the project in case of any side effect presented by the animals. Veterinarians were prepared to perform prompt evaluation and treatment of these animals in case of any pain, suffering or severe symptoms, and even to evaluate whether the animal should undergo a more specific treatment or even humanitarian euthanasia (with the owner’s consent), at which point animals were excluded from the study.

Overall survival time was defined as the time between surgical excision of the primary tumor and the date of death for each animal. Patients who died during the follow-up period were necropsied at the Veterinary Pathology Laboratory to determine the cause of death and to assess for possible metastasis.

### Statistical analysis

Data were classified as follows: tumor size (3.1–5 cm or > 5 cm), metastasis in the lymph nodes (yes or no), clinical stage (II, III, IV or V), histological grade (II or III), use of chemotherapy with carboplatin (yes or no), body condition score (obese, normal, slim), adjuvant treatment with LDN (yes or no), percentage of CD4+ and CD8+ lymphocytes (low or high), CD4+/CD8+ ratio (low or high) [15], and quality of life score (low or high) [16].

To evaluate the normality of the data distribution, results were initially subjected to the Kolmogorov-Smirnov test. For parametric and nonparametric data, Student’s t test/Mann-Whitney U test and one-way analysis of variance (ANOVA)/Kruskal-Wallis were used, respectively. Similarly, correlations were interrogated by Spearman tests. Survival curves were estimated and compared using the Kaplan-Meier method and log-rank test (Mantel-Cox).

All prognostic factors were analyzed by multivariate tests through analysis of hierarchical clusters, nonhierarchical cluster and principal component analysis. In all cases, statistical comparisons and tests were considered significant when *p* < 0.05. Analyses were performed using Prism 5.0 software (GraphPad, San Diego, CA, USA) or SPSS 17 software (SPSS Inc., Chicago, IL, USA).

## Results

### Clinical and pathological features

Age of female dogs in the study ranged from five to 18 years (mean = 11, median = 11.5), with a higher frequency of animals between 10 and 14 years (60%). The most affected breed was the poodle (50%, 15/30), followed by mixed breed dogs (20%, 12/60). The distribution of animals in the different groups with respect to their corporal score is shown in the Table 1. Only 10% (6/60) of dogs presented a body weight lower than that recommended for their age and breed, while 70% (42/60) showed normal weight parameters, and 20% (12/60) were considered obese.

**Table 1:** Distribution of frequencies by groups according to corporal score.

Experimental Groups	Corporal Score		
	0	1	2
MC-BMT(+) C + LDN	10.0% (6/60)	6.6% (4/60)	-
MC-BMT(+) C	10.0% (6/60)	3.3% (2/60)	3.3% (2/60)
MC-BMT(-) C + LDN	13.3% (8/60)	3.3% (2/60)	-
MC-BMT(-) C	10.0% (6/60)	3.3% (2/60)	3.3% (2/60)
MC-BMT(+)	13.3% (8/60)	3.3% (2/60)	-
MC-BMT(-)	13.3% (8/60)	-	3.3% (2/60)
**Total**	**70% (42/60)**	**20% (12/60)**	**10% (6/60)**

Score “0” corresponds to animals with normal weight, score “1” to animals with overweight, and “2” to animals that were below the normal weight for their age and breed.

Based on the reproductive history of study animals, the most frequently observed abnormality was pseudopregnancy in 20% (12/60) of the animals before the beginning of this study. Thirty percent (18/60) of the female dogs had already been treated with progestin to suppress estrus before the tumor diagnosis. Analysis of postsurgery biopsies with respective grading, size, affected lymph nodes and stage is presented in Table 2.

**Table 2:** Clinical and pathological parameters of female dogs with MC-BMT and correlation with the presence (+) or absence (-) of LDN treatment.

Parameters	LDN (-)	LDN (+)	*p*
Grading			
Grade II	16 (40%)	18 (45%)	0.531
Grade III	04 (10%)	02 (05%)	
Size			
3.1–5 cm	04 (10%)	06 (15%)	0.606
> 5 cm	16 (40%)	14 (35%)	
Lymph node**			
0	10 (25%)	10 (25%)	0.999
1	10 (25%)	10 (25%)	
Staging			
3	08 (20%)	10 (25%)	0.452
4	08 (20%)	10 (25%)	
5	04 (10%)	00 (0%)	
Quality of life***			
0	14 (35%)	02 (05%)	0.006*
1	06 (15%)	18 (45%)	
Survival			
To 180 days	14 (35%)	02 (05%)	0.006*
> 180 days	06 (15%)	18 (45%)	
Condition			
Live	02 (05%)	20 (100%)	0.000*
Dead	18 (45%)	00 (00%)	

*Significant differences at *p* < 0.05, using the Spearman statistical test

**0 –without metastasis; 1- presence of metastasis

***0–0 to 20 points in the scale proposed by [16]; 1–21 to 36 points in the same scale.

Regarding chemotherapy, 90% (36/40) of female dogs received six chemotherapy sessions, completing the planned cycle. Ten percent (4/40) of animals died after the fourth chemotherapy session, and all these female dogs presented a significant myelosuppression, which is a possible consequence of the chemotherapy treatment itself. Among possible side effects of chemotherapy, 10% (4/40) of female dogs exhibited vomiting after each chemotherapy session, and 5% (2/40) developed diarrhea. Among animals that did not receive LDN treatment, the distribution of VCOG-CTCAE grades for adverse effects were as follows: 80%, Grade 1, 15% Grade 2, and 5% Grade 3. None of the animals that exhibited adverse effects were subjected to LDN adjuvant treatment.

Based on hematological parameters, anemia and leukopenia were the most common findings, observed in both the control group and in the group submitted to chemotherapy without LDN (S1 Table). In contrast, in the two subgroups treated with LDN, animals maintained red and white blood cell and platelet counts within the normal range described for the species [17]. Biochemical profiles of the patients revealed higher creatinine values in the control group (S2 Table).

Among the 60 female dogs in the study, death was recorded in the control (12/20), MC-BMT(+) C (8/10), and MC-BMT(-) C (4/10) groups, primarily due to evolution of the neoplastic process (metastases and paraneoplastic syndromes) (18/24) and hypovolemic shock (6/24). Despite indication by the medical team, humane euthanasia was not performed because the owners chose not to perform this procedure. The animals were kept domiciled with pain control and under continuous supervision of the medical team until the moment of death. There were no deaths in the subgroups treated with LDN.

Necropsies were conducted in the 24 female dogs that died, revealing lesions indicative of metastasis, and it was subsequently confirmed that these metastases were consistent with the primary tumor by microscopic analysis. Some animals exhibited more than one metastatic site. Secondary sites included the lung (18/24), skin (10/24), liver (6/24) and kidney (4/24). In this study, 16 Grade II and 4 Grade III tumors were identified in the MC-BMT(-) group, and 18 Grade II and 2 Grade III tumors were identified in the MC-BMT(+) group.

Comparative analysis of clinical and pathological findings, quality of live and survival in LDN-treated and untreated female dogs are presented in Table 2. These data indicate that adjuvant treatment with LDN is statistically correlated to increased survival and improved quality of life.

### Immunophenotyping of peripheral blood lymphocytes

**Fig 1:**
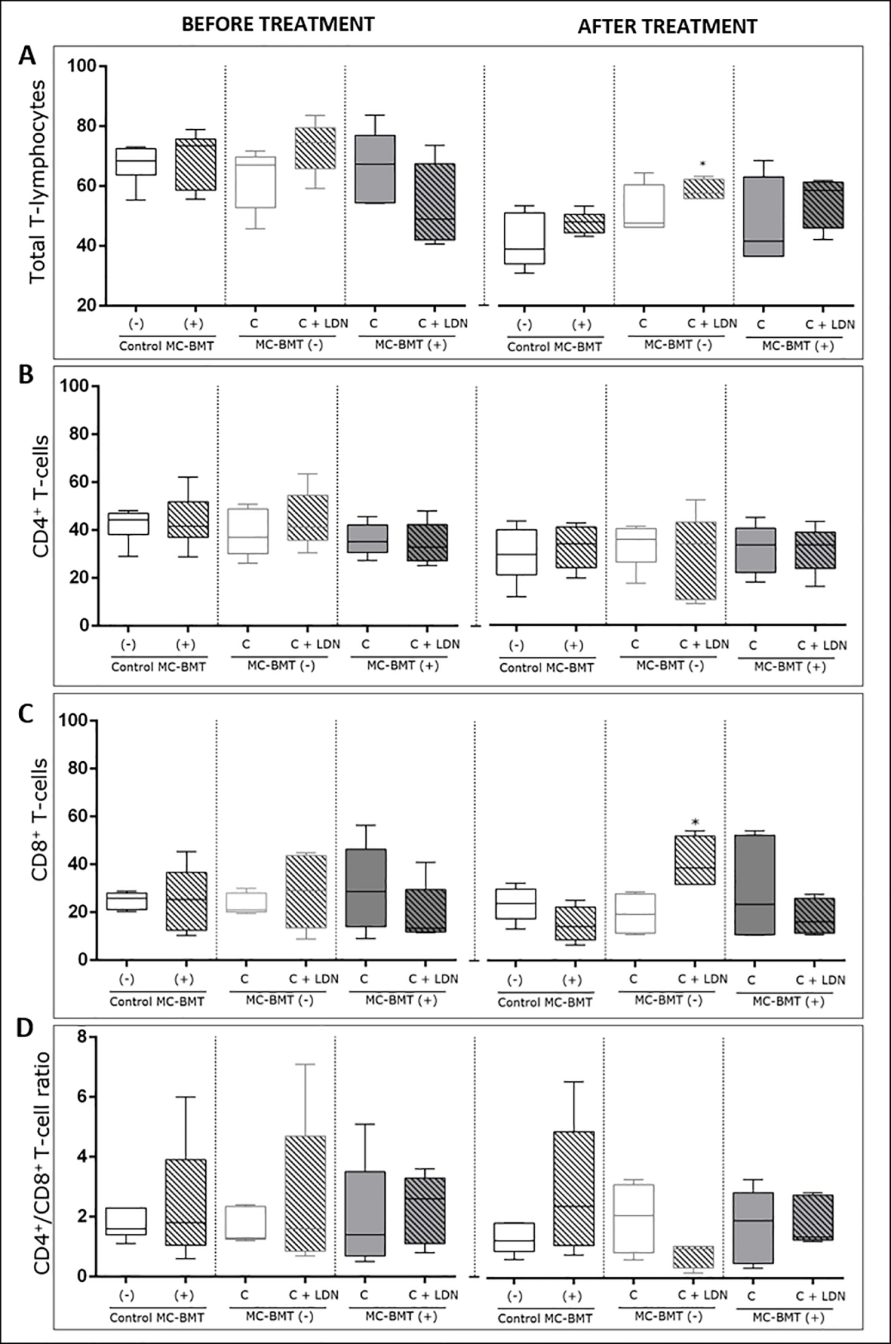
Flow cytometry immunophenotyping of total T-lymphocytes, CD4+ and CD8+ T-lymphocytes, and CD4+/CD8+ T-cell ratio in peripheral blood of female dogs with MC-BMT. Analysis is divided into before and after proposed treatment, subcategorized by the absence (-) or presence (+) of metastasis. Immunophenotypic analyses were performed using dual color flow cytometry as described in the Methods. Total lymphocytes were first selected based on their size (laser-forward scatter–FSC) and complexity (laser side scatter–SSC), and subsets were analyzed by quadrant statistics on FL1/FITC versus FL2/R-PE dot plots. Results are shown in a box plot format showing the minimum, median and maximum values of total T-lymphocytes. (A) CD4+ (B) and CD8+ T-lymphocytes (C) (expressed as a percentage of gated lymphocytes) and CD4+/CD8+ T-cell ratio (D) (expressed as a proportion). *Significant differences at *p* < 0.05, according to the Kruskal-Wallis test.

Posttreatment analysis revealed a significant increase in the percentage of total T-cells in the LDN-treated group compared to groups treated with chemotherapy alone and MC-BMT controls (Fig 1A). Results also revealed a higher number of lymphocytes in the MC-BMT(+) C + LDN group (*p* = 0.0994). Data analysis demonstrated no significant differences in the percentage of CD4+ T-cells among the groups (Fig 1B), either before or after treatment.

There were no significant differences in the percentage of CD8+ T-cells among groups in the initial period before treatment. However, unlike results observed for CD4+ T-cells, after experimental evaluation, the percentage of CD8+ T-cells was significantly higher in the MC-BMT(-) C + LDN group (*p* < 0.05) than in the group treated with chemotherapy alone (MC-BMT(-) C) (Fig 1C). While the CD4+/CD8+ T-cell ratio was numerically lower in the MC-BMT(-) C + LDN group, it did not attain statistical significance (Fig 1D).

### Endogenous opioid peptide quantification

**Fig 2:**
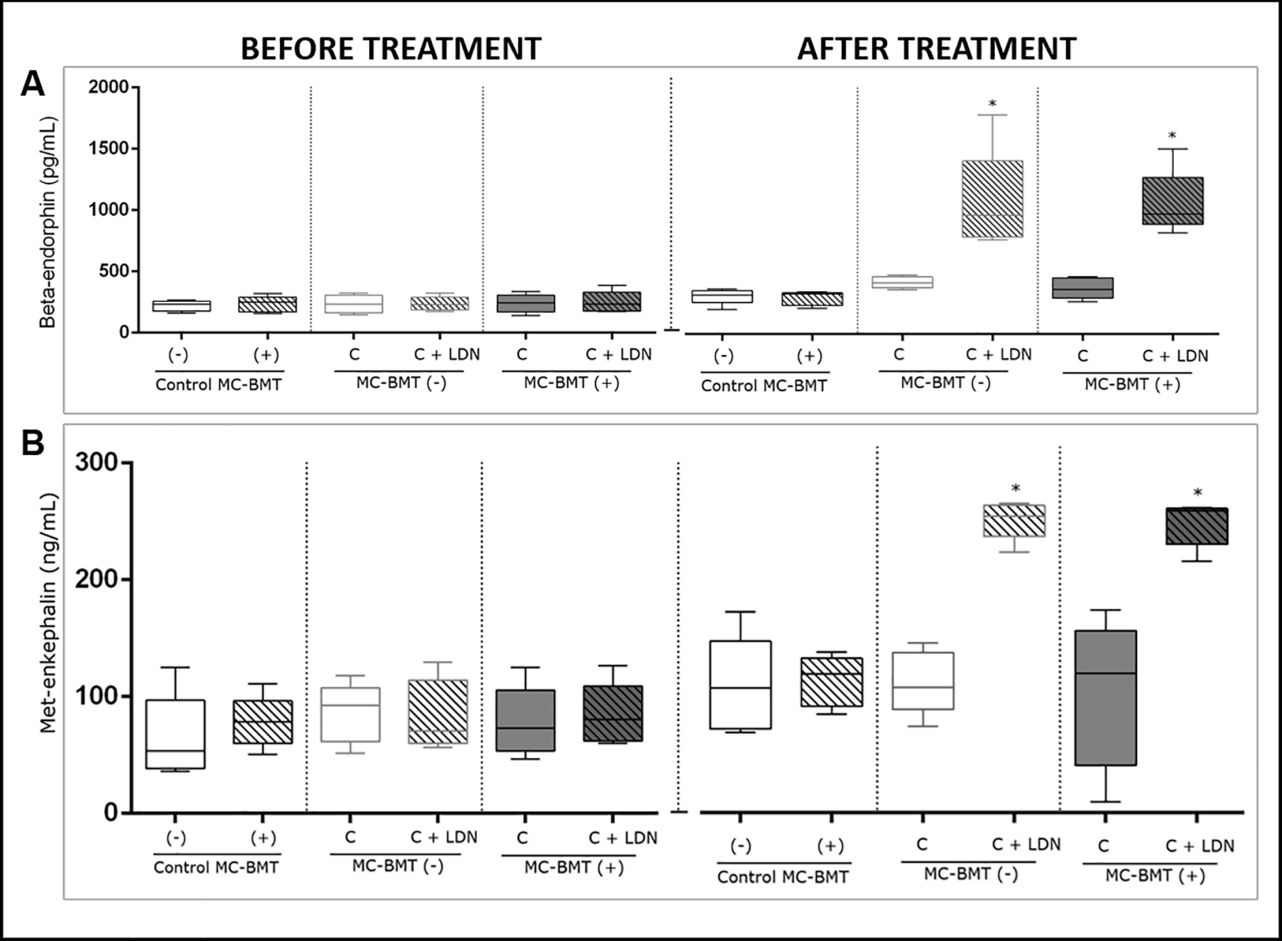
Beta-endorphin and met-enkephalin concentrations in the blood of female dogs with cancer as benign mixed tumors. The results are divided into before and after the proposed treatment and subcategorized by the absence (-) or presence (+) of metastasis. Beta-endorphin concentrations for each sample (in pg/mL) were determined by ELISA (A). Met-enkephalin concentrations for each sample were determined by ELISA and calculated in ng/mL (B). *Significant differences at *p* < 0.05, according to the Kruskal-Wallis statistical test.

All beta-endorphin serum concentrations were not significantly different among the experimental groups during the initial period. However, at the final assessment (after the last postmastectomy chemotherapy session), a significantly higher beta-endorphin concentration was observed in the MC-BMT(-) C + LDN and MC-BMT(+) C + LDN groups (*p* < 0.05) than in the MC-BMT(-) C and MC-BMT(+) C groups treated with chemotherapy alone (Fig 2A).

Regarding met-enkephalin serum analysis, no significant differences were observed among the groups during the initial period. However, at the end of the treatment, when compared to groups that were treated with chemotherapy alone, significantly higher concentrations of met-enkephalin were observed (*p* < 0.05) in groups where LDN was used as an adjuvant treatment (Fig 2B).

### Quality of life

**Fig 3:**
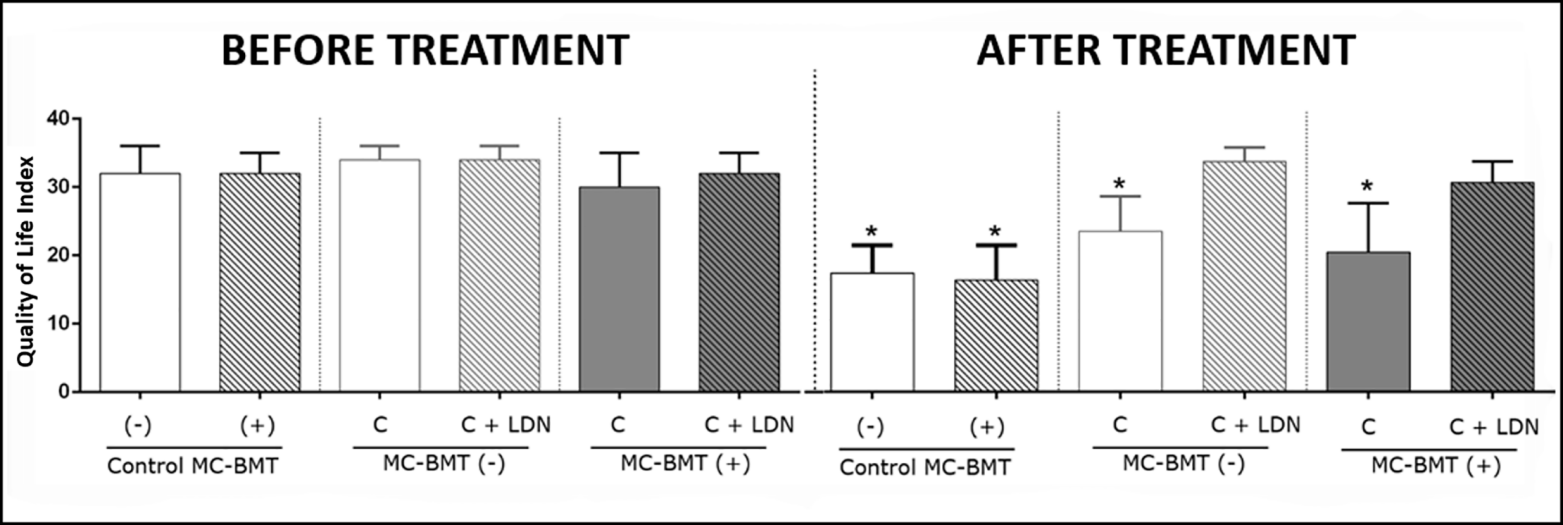
Representation of quality of life scores from female dogs with MC-BMT, before and after treatment with carboplatin, and in the presence of absence of LDN adjuvant treatment. *Significant differences at *p* < 0.05, according to the Kruskal-Wallis statistical test.

**Fig 4:**
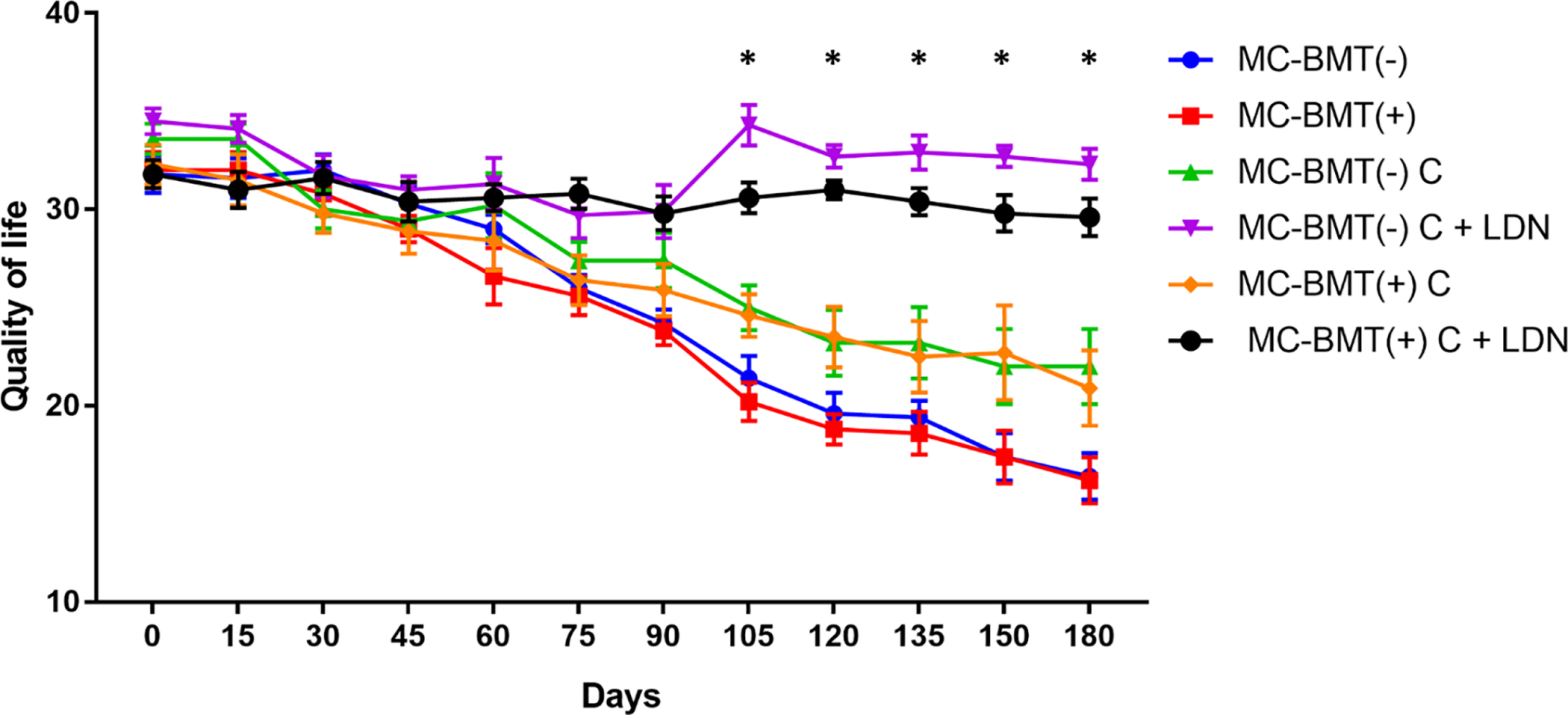
Temporal analysis on quality of life scores over time from female dogs with MC-BMT. Data are expressed as means of the quality of life scores for each group. *Significant differences between the LDN treated and LDN-not treated groups at *p* < 0.05. Statistical analysis was performed using a two-way ANOVA with post-hoc Tukey’s test.

Evaluation of quality of life data revealed that at the start of chemotherapy, all subgroup scores exhibited similar quality of life scores, ranging from 31 to 36 points (Fig 3). However, at the end of chemotherapy, groups treated with LDN, regardless of the presence or absence of metastasis, maintained their initial quality of life score, while groups treated with carboplatin only exhibited a significant reduction in quality of life, with scores ranging from 20 to 23 points. Female dogs in the control group showed initial quality of life scores similar to the group treated with chemotherapy, differing only at the end of the treatment, when scores ranged from 11 to 21 points. A temporal analysis of the quality of life scores showed that the LDN-treated groups presented statistically higher scores than the LDN-non treated groups at 105 days after the beginning of the experiment, and this difference was maintained until the end of the observation period (Fig 4).

When comparing groups based on sampling time, significant reductions were observed only for groups treated with carboplatin only. When the comparison was made only at posttreatment, there was a significant difference between the MC-BMT(-) C and MC-BMT(+) C groups (Table 3).

**Table 3:** Comparison of quality of life between experimental groups before and after the proposed treatment.

	Before treatment	After treatment	*p*-value (Wilcoxon test)
MC-BMT(-) C	34.0 (±2.5) ^Aa^	23.0 (±5.75) ^Ba^	0.0325*
MC-BMT(-) C+LDN	34.0 (±2.5) ^Aa^	33.0 (±2.25) ^Ab^	0.1250
p-value (Mann-Whitney)	> 0.9999	0.0079*	
MC-BMT(+) C	30.0 (±3.25) ^Aa^	20.0 (±5.75) ^Ba^	0.0345*
MC-BMT(+) C+LDN	32.0 (±2.25) ^Aa^	30.0 (±3.00) ^Ab^	0.1350
p-value (Mann-Whitney)	0.7381	0.0159*	

Capital superscript letters indicate *p* < 0.05 using the nonparametric Wilcoxon statistical test, when comparing the same group before and after treatment.

Lowercase superscript letters indicate *p* < 0.05 using the nonparametric Mann-Whitney statistical test, when comparing different groups at each time point.

*Significant differences at *p* < 0.05.

### Survival curve comparisons and statistical analysis

**Fig 5:**
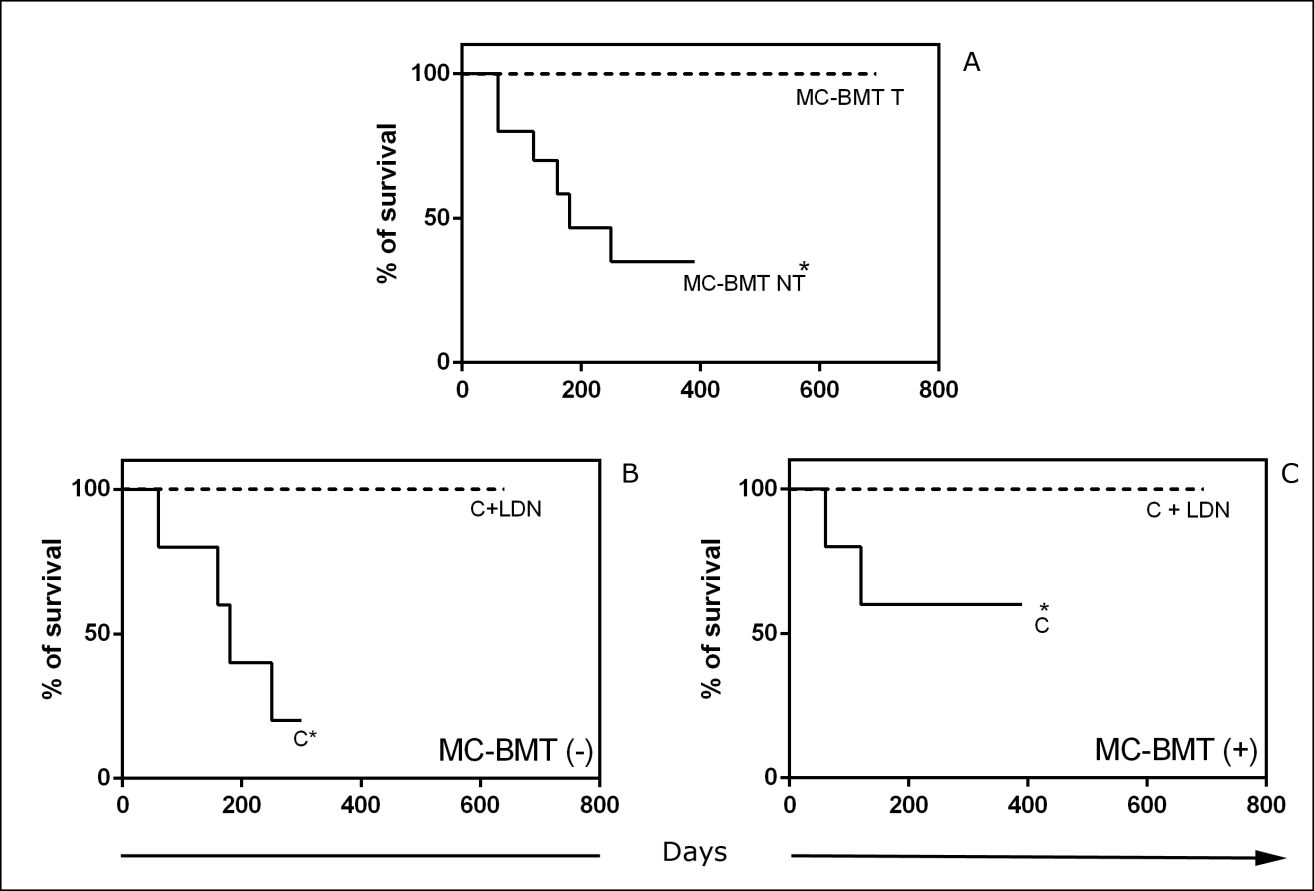
Survival rates of animals with MC-BMT. (A) Six-month survival curves of groups according to treatment (T) or not (NT) with LDN subgroups classified according to the absence (-) or presence (+) of lymph node metastasis (B and C). *Significant differences at *p*< 0.05 according to the Kaplan-Meier analysis.

Minimum survival time was 60 days after mastectomy, as seen in animals from the control group, which died due to lung metastasis, while a maximum of 695 days was observed in the group that underwent mastectomy and LDN-associated chemotherapy. Significant differences (*p* < 0.05) between groups treated and not treated with LDN were observed, regardless of the presence or absence of metastasis (Fig 5). The average observation times were 525.5, 200.2 and 123.1 days in groups treated with chemotherapy and LDN, chemotherapy alone and mastectomy only, respectively. Median survival was 180 and 150 days in groups treated with postoperative chemotherapy and mastectomy only, respectively. For the chemotherapy and LDN group, it was not possible to observe median survival because there were no deaths. The percent mortality was 60% in groups treated with postoperative chemotherapy and mastectomy only. There was no need to perform humanitarian euthanasia, as evaluated by the veterinary staff.

There was a significant association of survival (*p* < 0.0001 through both univariate and multivariate analysis) and quality of life after treatment (*p* = 0.004 on univariate analysis and *p* = 0.003 on multivariate analysis) with the use of LDN as an adjuvant treatment.

## Discussion

Mammary carcinomas in benign mixed tumors are the most common malignancies in female dogs [3], and there is a growing interest in studies directed at their biological behavior and treatment due to their high frequency and variable malignant potential. There was a higher frequency of animals diagnosed with MC-BMT in the 10 to 14-year age range. This result corroborates previously reported findings indicating an increasing frequency of mammary tumors in female dogs between nine and thirteen years [4, 18]. However, none of the studies in veterinary medicine have noted consistent differences in prognosis according to age [19].

According to Misdorp [1], the poodle breed has a marked genetic predisposition for the development of mammary tumors. However, Salas et al. [20] reported that, although mammary tumors are more frequently diagnosed in some breeds, there are insufficient data to support a breed predisposition for the development of this disease. In this study, similar to what was reported by Estrela-Lima [4], a higher frequency of poodle breed dogs with MC-BMT was observed. However, this fact may be associated with the high number of animals of this breed that are found in the study area or to the existence of several lineages and crossings [21].

Interesting and controversial results were observed regarding nutrition characteristics and corporal conditions of patients, because although the vast majority of the study animals (70%) had the presence of homemade food in their diet, the corporal condition was normal in most cases (70%). The findings in this study that most animals exhibited a normal corporal condition at the beginning of the experiment initially suggests an unmarked influence of this variable in tumor development; however, it is noteworthy that most obese female dogs (8/12) presented tumors larger than five centimeters as well as regional metastasis in an advanced stage. Thus, perhaps the dog corporal condition is an important factor for neoplastic progression and not for its initiation. Studies report that obese women have higher estrogen concentrations because of the transformation of androstenedione from the adipose tissue into estrone and then to estrogen, a hormone well known to be involved in breast carcinogenesis [22].

Average counts of erythrocytes, platelets and leukocytes observed in female dogs of the two subgroups that were not treated with LDN were lower compared to groups treated with this drug. The main side effect of carboplatin is an influence on the hematological profile, manifested primarily by leukopenia, anemia, and thrombocytopenia [23]. Moretti et al. [24] reported that carboplatin did not induce a significant decrease in hematological parameters when used as a chemotherapeutic in the treatment of carcinoma in 46 dogs; however, blood sampling in this study was conducted immediately after treatment. Thus, the decrease in leukocyte count observed in the present study may be attributed to the longer period of chemotherapy and prolonged observation of the animals.

Macroscopic data analysis of the tumors indicates the predominance of multicentric tumors with bilateral involvement of the mammary chain, and abdominal and inguinal mammary glands were the most affected; these tumors were usually larger than five centimeters. These results confirm those obtained by Sorenmo [25], which report that 57% of tumors are multicentric. Lana et al. [26] stated that the frequency of mammary carcinoma is significantly higher in inguinal and abdominal mammary glands but did not provide any explanation this predilection. According to Misdorp [1], these glands are the most affected due to increased mammary gland parenchyma, which can exhibit a greater proliferative change in response to hormones.

In this study, the majority of female dogs completed chemotherapy treatment; however, 40% of female dogs displayed vomiting after each chemotherapy session, and 10% had diarrhea. These side effects after the use of carboplatin are already described in the literature [27]. It is noteworthy that the vast majority of animals showing side effects (90%) did not receive LDN adjuvant treatment.

Naltrexone can be orally administered to animals and is low in cost, which facilitates its use. In humans treated with chemotherapy, low-dose naltrexone treatment promotes a decrease in side effects induced by chemotherapy [9, 28]. According to Metze et al. [12], LDN side effects in humans included lethargy, dizziness, vomiting, and diarrhea, but these conditions were not frequent and were limited to the start of treatment. In this study, patients that used LDN as adjuvant treatment did not show any of the side effects described above. These data are consistent with those from the study by Brown and Panksepp [28], which associated the use of LDN with quality of life and well-being in humans. By reducing chemotherapy side effects and maintaining quality of life, LDN may represent an important tool for adjuvant treatment of mammary tumors in dogs.

The percentage of CD8+ T-lymphocytes and beta-endorphin and enkephalin serum concentrations were significantly higher in the groups treated with LDN than in the non-LDN-treated and control groups; however, this increase in CD8+ T-cells was restricted to the group without metastases. Thus, it is believed that the LDN therapy provides a cellular immune response stimulus via CD8+ T-lymphocytes, and the absence of metastasis favors this benefit. In LDN-treated animals, there was a direct correlation between beta-endorphin and enkephalin concentrations and a higher CD8+ T-lymphocyte count. This result can be explained by the fact that lymphocytes express receptors for endogenous opioid peptides [29]. When used at low doses, naltrexone reversibly binds to endogenous opioid receptors for a short period. After three to four hours, naltrexone turns off these receptors, increasing beta-endorphin and enkephalin concentrations. Activation of these opioid peptide receptors in lymphocytes results in increased contact between the effector cells and tumor cells [30]. Additionally, through this contact, there is release of ATP, which reacts with the adenylate cyclase enzyme, consequently increasing cAMP in lymphocytes [31]. In turn, cAMP promotes selective activation of cytokines (IL-2 and IFN-γ) [32]. IFN-γ stimulates macrophages to secrete interleukin-2 (IL-2), which stimulates T-cells to proliferate and produce more IFN-γ [29]. Activated macrophages produce TNF-α, which promotes apoptosis of malignant cells [33].

In this study, beta-endorphin and enkephalin serum concentrations were significantly greater in the groups treated with LDN than in the groups not treated with LDN and the controls. These groups maintained a higher quality of life and exhibited increased survival compared to control and non-LDN-treated groups. These data reinforce the notion that endogenous opioid peptides involved in the metabolism of naltrexone are involved in well-being, providing an improvement in quality of life and patient survival as well as beneficial effects via stimulation of the immune system [28].

The results of this study support that quality of life is directly related to the beneficial effects of LDN, being important in the evolution of treatment and consequent survival. These data are consistent with those found by Berkson et al. [34] and Donahue et al. [9], which studied the use of naltrexone in humans with pancreatic and ovarian tumors, respectively. In those works, the use of naltrexone as an adjuvant treatment provided improved quality of life for human patients.

Comparison of survival curves demonstrated enhanced results in the groups treated with LDN compared to the groups not treated with LDN, regardless of the presence or absence of metastasis. These results are consistent with observations by Brown and Panksepp [28], who found that LDN treatment is directly linked to increased survival and to the maintenance of quality of life in humans. The effects of naltrexone adjuvant treatment reduced the side effects of chemotherapy in a human patient [34] and presented significant influence in the immune system of rats [9], as seen in the present work.

## Conclusions

This study represents the first description of naltrexone use in veterinary medicine as a chemotherapy-adjuvant treatment in female dogs with mammary carcinoma. Results of this study demonstrate that naltrexone reduces the side effects related to carboplatin chemotherapy. Naltrexone treatment increased beta-endorphin and met-enkephalin serum concentrations, improved the animals’ well-being, maintained their quality of life, and contributed to an increased survival rate in dogs undergoing chemotherapy, thus making LDN adjuvant treatment an important tool in the clinical management of mammary tumors in female dogs.

## Supporting information

S1 Table Hematologic parameters in female dogs with carcinoma in benign mixed tumor, stratified as before and after the proposed treatment. https://figshare.com/articles/Hematologic_parameters_in_female_dogs_with_carcinoma_in_benign_mixed_tumor_stratified_as_before_and_after_the_proposed_treatment/6977744.
(PDF)

S2 Table Serum biochemical parameters in female dogs with carcinoma in benign mixed tumor, stratified as before and after the proposed treatment. https://figshare.com/articles/Serum_biochemical_parameters_in_female_dogs_with_carcinoma_in_benign_mixed_tumor_stratified_as_before_and_after_the_proposed_treatment/6977747.
(PDF)
