# Supplementary material for: The effect of naltrexone as a carboplatin chemotherapy-associated drug on the immune response, quality of life and survival of dogs with mammary carcinoma
Source: PLoS One. 2018 Oct 4;13(10):e0204830. doi: 10.1371/journal.pone.0204830 (PMC6171873; doi:10.1371/journal.pone.0204830)
Supplement: S1 Table — https://figshare.com/articles/Hematologic_parameters_in_female_dogs_with_carcinoma_in_benign_mixed_tumor_stratified_as_before_and_after_the_proposed_treatment/6977744. (PDF) [file pone.0204830.s001.pdf]

S1 Table. Hematologic parameters in female dogs with carcinoma in benign mixed tumor, stratified as before and after the proposed treatment.

Hematological Parameter		Before					
		MC-BMT(-)	MC-BMT(+)	MC-BMT(-) C	MC-BMT(-) C + LDN	MC-BMT(+) C	MC-BMT(+) C + LDN
Red Blood Cells[a]		6,5±1,1	7,3±0,6	7±0,7	6,4±0,7	7,2±1.6	6±0,7
Platelets[b]		288±242.5	325±83	393±94.7	500±115.2	396±110.7	289±65.7
White Blood Cells[c]	Total	18.1±4.4	12±3.4	10.8±4	14.9±6.4	8.3±1.5	14.2±5
	Rods	0±368	0±45	0±27	0±461	0±27	0±147
	Segmented	11.8±4.6	8.7±3.1	8.9±3.5	8.3±3.9	5.9±0,9	10.7±4.5
	Eosinophils	0,7±0,5	0,7±0,4	0,7±0,4	0,5±0,2	0,2±0,4	0,8±0,2
	Monocytes	0,9±0,4	0,6±0,1	0,4±0,5	0,8±1	0,4±0,2	0,4±0,3
	Limphocytes	1.5±1.3	1.4±0.2	1.7±1.3	1.2±0.5	1.5±0.7	1.7±0.7
Hematological Parameter		After					
		MC-BMT(-)	MC-BMT(+)	MC-BMT(-) C	MC-BMT(-) C + LDN	MC-BMT(+) C	MC-BMT(+) C + LDN
Red Blood Cells[a]		4.4±0,6*	3,7±1,2*	3,9±0,7*	6.5±0,5	3,9±1,3*	6.7±0,5
Platelets[b]		212±76.7	170±125	48±22.3*	280±95.5	25±29.5*	364±136.5
White Blood Cells[c]	Total	25±7.2	32.9±6.1*	2.6±0,27*	6±0,45	1.4±1.3*	6.3±2.77
	Rods	500±136	500±429*	0±18	0±52,2	0±131	0±60
	Segmented	14.7±3.9	1.9±5.3*	3.5±1.2*	5.3±1.7	0,9±5.7	6±2.3
	Eosinophils	0,8±0,3	2±0,8	0,07±0,1*	0,4±0,2	0,05±0,06*	0,3±0,2
	Monocytes	0,7±0,7	1,2±0,2	0,07±0,1*	0,1±0,2	0,03±0,01*	0,1±0,2
	Limphocytes	2.4±0.5	2.5±2.4	0.8±0.7	1.9±0.5	0.2±0.4	1.7±0.3

a Red blood cells are expressed as number x $10^6/mm^3$ of blood.
b Platelets are expressed as number x $10^3/mm^3$ of blood.
c White blood cells subpopulations are expressed as number x $10^3/mm^3$ of blood.
*Significant diferences at p<0,05.
