# Supplementary material for: The effect of naltrexone as a carboplatin chemotherapy-associated drug on the immune response, quality of life and survival of dogs with mammary carcinoma
Source: PLoS One. 2018 Oct 4;13(10):e0204830. doi: 10.1371/journal.pone.0204830 (PMC6171873; doi:10.1371/journal.pone.0204830)
Supplement: S2 Table — https://figshare.com/articles/Serum_biochemical_parameters_in_female_dogs_with_carcinoma_in_benign_mixed_tumor_stratified_as_before_and_after_the_proposed_treatment/6977747. (PDF) [file pone.0204830.s002.pdf]

S2 Table. Serum biochemical parameters in female dogs with carcinoma in benign mixed tumor, stratified as before and after the proposed treatment

Biochemical Parameter	Before					
	MC-BMT(-)	MC-BMT(+)	MC-BMT(-) C	MC-BMT(-) C + LDN	MC-BMT(+) C	MC-BMT(+) C + LDN
Urea[a]	49,1±23,7	42,6±6,4	42,2±5,8	44,7±10,7	41±0,9	54±10,4
Creatinine[a]	1±0,22	0,9±0,2	1±0,07	0,9±0,15	1±0,05	1±0
ALT[b]	44,9±10	42,3±23,7	33±23,4	28,8±5,25	58±42,8	51,2±14
AP[b]	159,2±47,1	183,8±92,3	160±65,2	119,4±35,9	160±50,6	159,2±941
Calcium[a]	12±2,6	13,3±2,2	13,2±2,6	13±3,4	14,4±16,5	9±2,9
Glucose[a]	85±5,9	87±15,6	83±11,5	87±16,3	104,8±29,2	73,5±34
**Biochemical Parameter**	**After**					
	MC-BMT(-)	MC-BMT(+)	MC-BMT(-) C	MC-BMT(-) C + LDN	MC-BMT(+) C	MC-BMT(+) C + LDN
Urea[a]	33±8,8	45,2±0,3	45,2±0,3	45,2±0,7	45,3±0,7	45,2±3,4
Creatinine[a]	1,±0,15	<u>1,4±0,2*</u>	1±0,35	1,1±0,1	1±0,1	1±0,1
ALT[b]	51±33,2	29,2±1,6	29,2±1,6	27,6±0,8	29,2±2	29,2±17,6
AP[c]	125,8±57,6	112,9±17,8	125,8±16,8	99,9±12,9	99,9±16,9	99,9±16,9
Calcium[a]	9,7±0,9	9±0,6	9±0,5	10,4±0,7	9,7±0,7	9±0,7
Glucose[a]	90±8,2	87±6,5	87±9	110±11,5	100±11,5	100±11,5

a Urea, creatinine, calcium, and glucose are expressed as mg/dL of blood.
b ALT is expressed as UI/dL of blood.
c Alkaline phosphatase (AP) is expressed as U/dL of blood.
*Significant diferences at *p*<0,05.
